# Tight Neck-Clothing

**Published:** 1910-05-14

**Authors:** Walter G. Walford

**Affiliations:** 120 Finchley Road


*To the Editor of* The Hospital.

### TIGHT NECK-CLOTHING.

*To the Editor of* The Hospital.

Dear Sir,—With reference to my pamphlet on the effects of cerebral congestion and tight neck-clothing that you have just reviewed, your reviewer hints in very courteous terms what one or two others have stated rather more abruptly, that while there may be something in it, yet that I claim a little too much. I showed a rather sarcastic review to a layman who had had good reasons for knowing that I was not overstating, and it may interest you to hear how he reviewed the reviewer. You quite gather that I am not alluding to the review in your journal. My friend said, "Well, the reviewer admits that you are possibly right, but then he says that you are claiming too much, and then he goes on to say that you are drawing attention to a possible cause of certain abnormal conditions which he admits have not been investigated; how in the world does he know whether you are right or wrong?—he quite gives himself away."

Now, sir, what I would suggest is, why should not you make a superficial investigation into this matter? You could easily in half an hour see if I have a *prima facie* case or not. I could come myself—you would very soon see that I am a little different from the average broken-down gouty man of nearly seventy-two, and I could bring with me four or five now healthy people in good position, formerly sufferers, whose evidence you could hardly question. Because, if I have not said too much, and I know that I have not done so, the sooner this matter is thrashed out the better.

I hold two letters from medical men that I have received in consequence of my pamphlet, stating the experience that they have had of my views in their own persons. Every day adds to my facts. One tells me that over thirty years ago he had a hard tumour at the root of his neck which interfered with his insuring his life, and that after consideration he loosened his neck-things, with the result that the tumour vanished in four months and he was enabled to insure at ordinary rates. When you come to think that in addition to the large arteries, veins, and nerves in the anterior triangles you have four secondary nerves, a network of sympathetic nerves, the trachea, thyroid, external jugular, and last but by no means least a heap of lymphatics, there is plenty of material for a tight neck-band to disarrange. Major Thurston, who at the Millbank Military Hospital has to some extent investigated the matter, says that it is "a very wide field and requires a regular commission to investigate it properly." Indeed, sir, you would be astonished to find how far it goes and how many old ideas will be upset when it is thrashed out.—Yours faithfully,

Walter G. Walford.

120 Finchley Road,
April 17.

